# On a Relation Between Packing and Covering Densities of Convex Bodies

**DOI:** 10.1007/s00454-019-00121-x

**Published:** 2019-08-26

**Authors:** Roman Prosanov

**Affiliations:** 1 Université de Fribourg, Chemin du Musée 23, 1700 Fribourg, Switzerland; 2 Moscow Institute of Physics and Technology, Institutskiy per. 9, Dolgoprudny, Russia 141700; 3 Technische Universität Wien Freihaus, Wiedner Hauptstraße 8-10, 1040 Wien, Austria

**Keywords:** Packings, Coverings, The Kuperberg conjecture

## Abstract

We show that a convex body admits a translative dense packing in $\mathbb {R}^d$ if and only if it admits a translative economical covering.

## Introduction

### Packing and Covering Densities

Let *C* be a *d*-dimensional convex body in $\mathbb {R}^d$, i.e., a compact convex set with nonempty interior. A (translative) *arrangement* is a set $C+A$, where *A* is a discrete point set in $\mathbb {R}^d$. We assume that *A* is infinite. An arrangement is called *packing* if no two translates of *C* in $C+A$ have an interior point in common. An arrangement is called *covering* if $\mathbb {R}^d = C+A$.

Define *upper* and *lower* densities of an arrangement

$$\begin{aligned} \overline{\mathrm{den}}(C+A)= & {} \limsup _{r \rightarrow \infty } \frac{\sum _{a \in A}\text {vol}((C+a)\cap B^d(r))}{\text {vol}(B^d(r))}, \\ \underline{\mathrm{den}}(C+A)= & {} \liminf _{r \rightarrow \infty } \frac{\sum _{a \in A}\text {vol}((C+a)\cap B^d(r))}{\text {vol}(B^d(r))}, \end{aligned}$$

where $B^d(r)$ is the Euclidean ball of radius *r* centered at the origin.

The (translative) *packing density* of *C* is

$$\begin{aligned} \delta _T(C) = \sup _{C+A \mathrm{~is~a~packing}} \overline{\mathrm{den}}(C+A). \end{aligned}$$

Similarly, the (translative) *covering density* of *C* is

$$\begin{aligned} \theta _T(C) = \inf _{C+A \mathrm{~is~a~covering}} \underline{\mathrm{den}}(C+A). \end{aligned}$$

An important example is a *periodic arrangement*, i.e., an arrangement of the form $C+\Lambda + X$, where $\Lambda$ is a lattice and *X* is a finite point set. In this case,

$$\begin{aligned} \overline{\mathrm{den}}(C+\Lambda + X)=\underline{\mathrm{den}}(C+\Lambda + X) = \frac{|X|\text {vol}(C)}{\text {vol}(\mathbb {R}^d/\Lambda )}. \end{aligned}$$

Then we will denote this quantity just as $\mathrm{den}(C+\Lambda + X)$.

We can consider arrangements consisting not only from translates of *C*, but from any congruent copies of *C*. In this case the packing and covering densities of *C* can be defined similarly. Denote them by $\delta (C)$ and $\theta (C)$ respectively. Another important case is the case of *lattice arrangements*, i.e., of the form $C+\Lambda$, where $\Lambda$ is a lattice. The corresponding densities over lattice arrangements only are denoted by $\delta _L(C)$ and $\theta _L(C)$.

Bounding packing and covering densities (especially for some specific choices of *C*, e.g., Euclidean balls) is one of the main problems in discrete geometry. Despite a lot of progress, plenty important questions remain open.

Clearly, for any *C* we have

$$\begin{aligned} \delta _T(C) \leqslant \delta (C) \leqslant 1 \end{aligned}$$

and

$$\begin{aligned} \theta _T(C) \geqslant \theta (C) \geqslant 1. \end{aligned}$$

The equality of any of these densities to 1 is equivalent to the property that copies of *C* can tessellate $\mathbb {R}^d$. This was proved by Schmidt [19], one can also look for a proof in [5, p. 805].

$$\begin{aligned} \delta (C)= & {} 1~~~~~~\Longleftrightarrow ~~~~~~\theta (C)=1, \\ \delta _T(C)= & {} 1~~~~~~\Longleftrightarrow ~~~~~~\theta _T(C)=1. \end{aligned}$$

A natural question arises from this observation: if a body *C* cannot be packed densely, does it mean that it cannot cover $\mathbb {R}^d$ economically? In the book by Brass et al. [1] this conjecture is attributed to W. Kuperberg (Conjecture 1 in Chapter 1.10, the original notation is saved):

#### Conjecture 1.1

Let $d \geqslant 2$ be fixed. Then for any $\varepsilon > 0$ there exists a $\delta > 0$ with the property that for every *d*-dimensional convex body *C*,

$$\begin{aligned}&(1)~~~~~\delta (C) \leqslant 1-\varepsilon ~~~~~~{ {implies}}~~~~~~\theta (C) \geqslant 1 + \delta , \\&(2)~~~~~\theta (C) \geqslant 1+\varepsilon ~~~~~~{ {implies}}~~~~~~\delta (C) \leqslant 1 - \delta . \end{aligned}$$

In the notation of Conjecture 1.1, $\delta$ and $\delta (C)$ are not the same. The notation $\delta (C)$ is conventional for the packing density and another $\delta$ is common for the epsilon–delta notation.

The aim of the present note is to prove this conjecture for translative densities. It will be more helpful to give our statement in the form of converse implications.

#### Theorem 1.2

Let $d \geqslant 2$ be fixed.

- Let $0< \varepsilon \leqslant {1}/{d^{d+1}}$ and *C* be a *d*-dimensional convex body, or ${0<\varepsilon <1}$ and *C* in addition be centrally symmetric. If for the translative packing density we have $\delta _T(C) > 1 - \varepsilon$, then the translative covering density of *C* satisfies
  $$\begin{aligned} \theta _T(C) < \big (1+\varepsilon ^{{1}/({d+1})}\big )^{d+1}. \end{aligned}$$
- Let ${1}/{d^{d+1}}< \varepsilon < 1$ and *C* be non-centrally symmetric. If for the translative packing density we have $\delta _T(C) > 1 - \varepsilon$, then the translative covering density of *C* satisfies
  $$\begin{aligned} \theta _T(C) < \big (1+\varepsilon d^d\big )\left( 1+\frac{1}{d}\right) ^d. \end{aligned}$$
- Let $0< \varepsilon <1$ and *C* be a *d*-dimensional convex body. If for the translative covering density we have $\theta _T(C) < 1 + \varepsilon$, then the translative packing density of *C* satisfies
  $$\begin{aligned} \delta _T(C) > \big (1-\varepsilon ^{{1}/({d+1})}\big )^{d+1}. \end{aligned}$$

Clearly, Conjecture 1.1 for translative densities follows from this theorem.

### Previous Results and Future Perspectives

The only already known case of Conjecture 1.1 was established by Ismailescu in [8]. He considered $d=2$ and only centrally symmetric convex bodies. More precisely, he showed that in this case

$$\begin{aligned} 1-\delta _L(C) \leqslant \theta _L(C) -1 \leqslant 1.25\sqrt{1-\delta _L(C)}. \end{aligned}$$

Fejes Tóth [6] established that for any planar centrally symmetric convex body *C* we have $\delta (C)=\delta _T(C)=\delta _L(C)$, and Dowker [2] proved that $\theta _L(C) = \theta _T(C)$. Hence, Ismailescu’s result extends to the case of all translative arrangements. His proof is based on the approximation of *C* by centrally symmetric octagons and cannot be extended to higher dimensions.

In [4] Fejes Tóth and Kuperberg proposed to understand links between packing and covering densities in a more general way. They defined the set $\Omega _d$ (resp. $\Omega ^*_d$) of points $(x, y) \in \mathbb {R}^2$ such that there exists a *d*-dimensional convex (resp. centrally symmetric) body *C* with $\delta (C)=x$ and $\theta (C)=y$. The definition of $\Omega _d$ (resp. $\Omega ^*_d$) can be restricted to the case of translative or lattice densities. It may be of interest to characterize these sets. In fact, it is still unknown whether these sets are closed (but it is known for translative or lattice cases) or convex. We refer the reader to the paper [11] investigating the planar case. Several inequalities involving both $\delta _L(C)$ and $\theta _L(C)$ were established, e.g., in [9, 10, 21], but also only in low dimensions.

In order to prove the translative Kuperberg conjecture it is enough to consider only sufficiently small values of $\varepsilon$ with respect to *d*. When $\varepsilon$ is not very small and *d* is sufficiently large it is interesting to compare Theorem 1.2 with the best known general bounds on packing and covering densities.

In the case of coverings by translates of an arbitrary *d*-dimensional convex body *C* the following inequality was established by Fejes Tóth [3] (which slightly improves a previous result by Rogers):

$$\begin{aligned} \theta _T(C) \leqslant d\ln d+ d\ln \ln d + d +o(d). \end{aligned}$$

We see that Theorem 1.2 gives us a stronger bound if

$$\begin{aligned} 1-\delta _T(C) <\frac{\ln d}{ed^{d-1}} \end{aligned}$$

or if

$$\begin{aligned} 1-\delta _T(C) < \left( \frac{\ln (d \ln d +d\ln \ln d+d)}{d+1}\right) ^{d+1} \end{aligned}$$

and *C* in addition is centrally symmetric.

For packing densities of centrally symmetric convex bodies the following result by Schmidt [20] is the best known:

$$\begin{aligned} \delta _T(C)\geqslant \delta _L(C) \geqslant \frac{d\ln 2}{2^{d+1}} - o(1). \end{aligned}$$

Comparing with the last inequality in Theorem 1.2 we see that the latter gives a stronger bound for sufficiently large *d* if

$$\begin{aligned} \theta _T(C)-1< \left( \frac{1}{2}- \frac{\ln (d\ln 2)}{d+1}\right) ^{d+1}. \end{aligned}$$

For a non-centrally symmetric *C* we should use the observation of Minkowski (see [7, Chap. 2]):

$$\begin{aligned} \delta _T(C)=2^d\delta _T(C-C)\frac{\text {vol}(C)}{\text {vol}(C-C)}. \end{aligned}$$

Denote $\big (\tfrac{\text {vol}(C-C)}{\text {vol}(C)}\big )^{1/d}$ by *W*(*C*). Then we have

$$\begin{aligned} \delta _T(C) \geqslant \frac{cd}{W^d(C)}. \end{aligned}$$

Hence, we obtain a better bound provided *d* is sufficiently large and

$$\begin{aligned} \theta _T(C)-1< \left( 1 - \frac{1}{W(C)}-\frac{\ln \left( W(C)d\ln \sqrt{2}\right) }{d+1}\right) ^{d+1}. \end{aligned}$$

An interesting question is to understand if the dependence of our bounds on *d* is necessary. In other words, we would like to propose the following problem:

#### Question 1.3

Is it true that for any $\varepsilon > 0$ there exists $\mu > 0$ with the property that for every *d* and every *d*-dimensional convex body *C*,

$$\begin{aligned}&(1)~~~~~\delta (C) \leqslant 1-\varepsilon ~~~~~~{ {implies}}~~~~~~\theta (C) \geqslant 1 + \mu , \\&(2)~~~~~\theta (C) \geqslant 1+\varepsilon ~~~~~~{ {implies}}~~~~~~\delta (C) \leqslant 1 - \mu . \end{aligned}$$

It is also of interest to prove an analogue of Theorem 1.2 for the case of lattice densities only (it is conjectured [1] that in higher dimensions $\delta _L(C) \ne \delta _T(C)$ and $\theta _L(C) \ne \theta _T(C)$). Such a proof should use totally different ingredients.

Our proof is based on a bound on the number of steps of a certain greedy algorithm. It seems that in previous years most of results on packing and covering densities in higher dimensions used averaging or probabilistic arguments. Recently the focus started to shift in the direction of more deterministic techniques. For instance, in [12] Naszódi gave a new proof of some well-known covering results via discretization and a lemma connecting fractional covering numbers of finite hypergraphs with integral ones. Proofs of this lemma considered a greedy algorithm applied to finite sets. In [18] Rolfes and Vallentin explored a greedy algorithm applied directly to geometric covering problems and also obtained several classical results through their method.

Packing and covering results have some applications to other problems in discrete geometry. For instance, consider the problem of finding the *chromatic number* $\chi (S)$ of a subset $S \subseteq \mathbb {R}^d$. The chromatic number $\chi (S)$ is the minimal number of colors sufficient to color *S* in such a way that any two points at the distance 1 have different colors. Using deterministic covering algorithms in [13] the author gave a new proof of the upper bound for $\chi (\mathbb {R}^d)$ and in [14] the author established new upper bounds for $\chi (S^{d-1}_R)$, where $S^{d-1}_R$ is a $(d-1)$-dimensional Euclidean sphere of radius *R*. For more details about geometric chromatic numbers the reader is refereed to the surveys [15, 16].

## Proof of Theorem 1.2

We need an important theorem of Rogers (see [17, Thms 1.7, 1.9]).

### Theorem 2.1

For a convex body *C* in the definition of its translative packing density we can take the supremum over only periodic arrangements. The same holds for its translative covering density.

We assume that *C* contains the origin in the interior. By $\lambda C + x$ we denote the image of *C* under the composition of the homothety with the center at the origin and the coefficient $\lambda$ and the translation by the vector *x*. Now we are able to give a proof of the main theorem.

### Proof of (1)

Assume that $\delta _T(C) > 1 - \varepsilon$. By Theorem 2.1 there is a lattice $\Lambda$ and a finite point set *X* such that $C+\Lambda +X$ is a packing and

$$\begin{aligned} \mathrm{den}(C+\Lambda +X) > 1 - \varepsilon . \end{aligned}$$

Consider the torus $T=\mathbb {R}^n/\Lambda$. The sets *X* and *C* can be projected to *T*. Abusing the notation, we still denote by *X* and *C* their images under this projection. This will not lead to an ambiguity as from now on we work only on *T*. The arrangement $C+X$ is a packing on *T*.

Let $$k=|X|$$. Then

$$\begin{aligned} \frac{k\,\text {vol}(C)}{\text {vol}(T)} = \mathrm{den}(C+\Lambda +X) > 1- \varepsilon . \end{aligned}$$

As our problem is homothety invariant, we may assume that $\text {vol}(T)=1$. Hence,

$$\begin{aligned} k\,\text {vol}(C) > 1-\varepsilon . \end{aligned}$$

Define $S_0=\text {vol}(T \backslash (C+X))$. We have $S_0 < \varepsilon$. Also, let $X_0=X$.

Fix $0< \alpha < 1$. We proceed iteratively. Assume that $\left( 1+\alpha \right) C + X_i$ does not cover *T*. Then there exists $y \in T$ which is not covered by $\left( 1+\alpha \right) C+X_i$. We have that for every $x \in X$,

$$\begin{aligned} \left( -\alpha C + y\right) \cap \left( C + x\right) = \emptyset . \end{aligned}$$

Indeed, $-\alpha C + y$ can be obtained as the image of $C + x$ under a homothety with the coefficient $-\alpha$ and the center in the segment *xy* laying outside of $C + x$. Next, we are looking for $y'$ such that $C+y'$ covers $-\alpha C + y$. If *C* is centrally symmetric, then we can take $y'=y$. In the other case we need the condition $\alpha \leqslant {1}/{d}$. Then the existence of such $y'$ follows from the fact that we can put a translate of $-\frac{1}{d}\,C$ into *C*. This statement is equivalent to the existence of a point in the interior of *C* such that every chord through this point is divided in a ratio not greater than *d*. It is a well-known implication of the Helly theorem and was proved by Minkowski and Radon, see, e.g., [22, Cor. 1.4.2].

Consider $X_{i+1} = X_i \cup \{y'\}$ and $S_{i+1} = \text {vol}(T \backslash (C+X_{i+1}))$. Clearly,

$$\begin{aligned} 0 \leqslant S_{i+1} \leqslant S_i - \text {vol}\left( -\alpha C\right) = S_i - \alpha ^d \text {vol}(C) < \varepsilon - (i+1)\alpha ^d\text {vol}(C). \end{aligned}$$

If $\left( 1+\alpha \right) C + X_{i+1}$ does not cover *T*, then repeat this process. At every step $S_i \geqslant 0$. Hence, we stop after *l* steps and the upper bound on *l* can be deduced from the inequality

$$\begin{aligned} \varepsilon > l \alpha ^d\,\text {vol}(C). \end{aligned}$$

We rewrite it as

$$\begin{aligned} l\,\text {vol}(C) < \frac{\varepsilon }{\alpha ^d}. \end{aligned}$$

We obtain that $\left( 1+\alpha \right) C + X_l$ covers *T*. Then $\left( 1+\alpha \right) C + X_l + \Lambda$ covers $\mathbb {R}^d$. Now we need to estimate the density of this arrangement

$$\begin{aligned} \theta _T(C)= & {} \theta _T(\left( 1+\alpha \right) C) \\\leqslant & {} \mathrm{den}\left( \left( 1+\alpha \right) C+X_l+\Lambda \right) = |X_l| \text {vol}\left( \left( 1+\alpha \right) C\right) \\= & {} (k+l)\left( 1+\alpha \right) ^d\text {vol}(C) = (k\,\text {vol}(C)+l\,\text {vol}(C))\left( 1+\alpha \right) ^d. \end{aligned}$$

As $C+X_0$ is a packing, then $k\,\text {vol}(C) \leqslant 1$. Using this and $l\,\text {vol}(C) < {\varepsilon }/{ \alpha ^d}$ we get

$$\begin{aligned} \theta _T(C) < \left( 1+\frac{\varepsilon }{ \alpha ^d}\right) (1+\alpha )^d. \end{aligned}$$

After the calculation of the derivative in $\alpha$ we can see that for fixed *d* and $\varepsilon$ this expression attains its minimal value at $\alpha =\varepsilon ^{{1}/({d+1})}$. If $\varepsilon ^{{1}/({d+1})} \leqslant {1}/{d}$, then it is an admissible value for any *C*. After the substitution we obtain the bound

$$\begin{aligned} \theta _T(C) < \Big (1+\varepsilon ^{\frac{1}{d+1}}\Big )^{d+1}. \end{aligned}$$

If $\varepsilon ^{\frac{1}{d+1}} > \frac{1}{d}$ and *C* is not centrally symmetric, then the admissible value of $\alpha$ minimizing the expression at the right-hand side is $\alpha =\frac{1}{d}$. In this case we have

$$\begin{aligned} \theta _T(C) < \big (1+\varepsilon d^d\big )\Big (1+\frac{1}{d}\Big )^d. \end{aligned}$$

$$\square $$

### Proof of (2)

Assume that $\theta _T(C) < 1 + \varepsilon$. Similarly, by Theorem 2.1 there is a lattice $\Lambda$ and a finite point set *X* such that $C+\Lambda +X$ is a covering and

$$\begin{aligned} \mathrm{den}(C+ \Lambda +X) < 1 + \varepsilon . \end{aligned}$$

Moreover, we can choose $\Lambda$ such that for any $\lambda _1$ and $\lambda _2 \in \Lambda$, the translate $C+\lambda _1$ does not intersect $C+\lambda _2$. Indeed, let $m > 0$ be an integer. For every element $\gamma$ of the group $\Lambda / m\Lambda$ choose a representative $\lambda (\gamma ) \in \Lambda$. By $\Lambda _m \subset \mathbb {R}^d$ denote the set $\{\lambda (\gamma ) : \gamma \in \Lambda /m\Lambda \}$. There exists *m* such that the desired condition is satisfied for $m\Lambda$. Take $X' = X + \Lambda _m$. Then $C+m\Lambda +X'$ is a covering and

$$\begin{aligned} \mathrm{den}(C+m\Lambda +X') = \mathrm{den}(C+\Lambda +X) < 1+ \varepsilon . \end{aligned}$$

Then we can replace $\Lambda$ with $m\Lambda$ and *X* with $X'$.

Let *T* be the torus $\mathbb {R}^n/\Lambda$ and $k=|X|$. As in the proof of (1) from now on we consider *X* and *C* as subsets of *T*. Then $C+X$ is a covering of *T* and

$$\begin{aligned} \frac{k\,\text {vol}(C)}{\text {vol}(T)} = \mathrm{den}(C+\Lambda +X) < 1+ \varepsilon . \end{aligned}$$

As previously, assume that $\text {vol}(T)=1$. Hence,

$$\begin{aligned} k\,\text {vol}(C) < 1+\varepsilon . \end{aligned}$$

Define $S_0=k\,\text {vol}(C)-1$. Then $S_0 < \varepsilon$. Also, let $X_0=X$.

Fix $0< \alpha < 1$. Now we proceed iteratively. Assume that $\left( 1-\alpha \right) C + X_i$ is not a packing. Then there exists $y \in T$ and $x, x' \in X_i$ such that

$$\begin{aligned} \left( \alpha C + y\right) \subset \left( (C+x)\cap (C+x') \right) . \end{aligned}$$

Indeed, there are $x, x' \in X_i$ such that

$$\begin{aligned} \left( \left( 1-\alpha \right) C+x\right) \cap \left( \left( 1-\alpha \right) C+x'\right) \ne \emptyset . \end{aligned}$$

Choose *y* in their intersection. Then clearly

$$\begin{aligned} \left( \alpha C + y\right) \subset \left( \alpha C + \left( 1-\alpha \right) C+x \right) = C+x. \end{aligned}$$

Similarly, $$\left( \alpha C + y\right) \subset C+x'$$.

Consider $$X_{i+1} = X_i \backslash \{x'\}$$ and

$$\begin{aligned} S_{i+1} =|X_{i+1}|\text {vol}(C)-(1-\text {vol}(T \backslash (C+X_{i+1}))). \end{aligned}$$

Naturally, $S_i$ measures the covering excess of the arrangement $C+X_i$. We obtain

$$\begin{aligned} 0 \leqslant S_{i+1} \leqslant S_i - \text {vol}\left( \alpha C\right) = S_i - \alpha ^d\text {vol}(C) < \varepsilon - (i+1)\alpha ^d \text {vol}(C). \end{aligned}$$

If $\left( 1-\alpha \right) C + X_{i+1}$ is not a packing, then repeat this process. At every step $S_i \geqslant 0$. Hence, as previously we will stop after *l* steps, where

$$\begin{aligned} l\,\text {vol}(C) < \frac{\varepsilon }{\alpha ^d}. \end{aligned}$$

We have $\left( 1-\alpha \right) C + X_l$ is a packing in *T* and $\left( 1-\alpha \right) C + X_l+\Lambda$ is a packing in $\mathbb {R}^d$. Now we need to estimate the density of this arrangement

$$\begin{aligned} \delta _T(C)= & {} \delta _T(\left( 1-\alpha \right) C)\geqslant \mathrm{den}\left( \left( 1-\alpha \right) C + X_l +\Lambda \right) \\= & {} |X_l| \text {vol}\left( \left( 1-\alpha \right) C\right) = (k-l)\left( 1-\alpha \right) ^d\text {vol}(C) \\= & {} (k\,\text {vol}(C)-l\,\text {vol}(C))\left( 1-\alpha \right) ^d > \left( 1-\frac{\varepsilon }{\alpha ^d}\right) \left( 1-\alpha \right) ^d. \end{aligned}$$

This expression is maximized as $\alpha = \varepsilon ^{{1}/({d+1})}$. Then we obtain

$$\begin{aligned} \delta _T(C)> \big (1-\varepsilon ^{{1}/({d+1})}\big )^{d+1}. \end{aligned}$$

$$\square $$
